# An Oxygenating Video Laryngoscope Blade With an Integrated Lens‑Clearing Gas Jet for Management of the Soiled Airway During Tracheal Intubation: Concept and Design Considerations

**DOI:** 10.7759/cureus.106392

**Published:** 2026-04-03

**Authors:** Kevin T Malone, Bailey L Jarrett, Andrew L Juergens, Dorian F Drigalla

**Affiliations:** 1 Emergency Medicine, Baylor Scott & White Medical Center - Temple, Temple, USA; 2 Emergency Medicine, Baylor College of Medicine, Temple, USA

**Keywords:** airway, apneic, decontamination, first‑pass, hematemesis, laryngoscope, laryngoscopy, oxygenation, soiled airway, video

## Abstract

In emergency and prehospital tracheal intubation, blood or emesis in the airway is common and is associated with reduced first‑pass success. Video laryngoscopy (VL) improves first‑attempt success in many critically ill populations but remains significantly vulnerable to lens occlusion.

Herein, we describe the conceptual design of a video laryngoscope (VL) blade that integrates a dedicated internal gas lumen terminating near the distal blade, where it can generate a high‑velocity jet directed across the camera lens. The primary intended function is real‑time lens clearing and lens‑occlusion prevention in the soiled airway. A secondary intended function is apneic oxygenation during laryngoscopy. A combined lens‑clearing and oxygen‑insufflating VL blade could reduce interruptions for lens cleaning, decrease repeated laryngoscopy attempts, and mitigate peri‑intubation hypoxemia in high‑risk scenarios such as massive hematemesis, trauma, and cardiac arrest.

We outline design requirements, key safety risks (including aerosol generation and pressure transients), prior art, and a staged validation plan comprising benchtop lens‑contamination testing, high‑fidelity simulation in standardized soiled‑airway scenarios, and subsequent clinical feasibility and effectiveness studies.

## Introduction

Securing an airway rapidly is often life‑saving, but emergent tracheal intubation carries substantial risk when performed in critically ill patients; particularly first-pass success (FPS) has been shown to be decreased by at least 10% in contaminated airways compared to non-contaminated airways [1]. First‑pass success is a key quality metric because multiple attempts are consistently associated with increased adverse events including hypoxemia, hypotension, aspiration, airway trauma, and cardiac arrest [1-4]. Intubations are performed through the use of a video laryngoscope (VL), utilizing a camera to visualize the vocal cords, or by utilizing a direct laryngoscope (DL), inserting the device to obtain direct visualization through line of sight.

A particularly high‑risk subset is the soiled airway, where blood, vomitus, or secretions impair ventilation and laryngoscopy. In emergency department (ED) intubations, soiled airways have been associated with reduced FPS using both direct laryngoscopy and VL [1]. It has been found that in a soiled airway, lens contamination negatively affects upwards of 15% of FPS with DL and VL technique [1]. In out‑of‑hospital cardiac arrest, emesis/regurgitation is commonly encountered (reported up to approximately one‑third in observational work), creating a recurrent failure mode for airway management during resuscitation [5].

Video laryngoscopy has improved glottic visualization and first‑attempt success in many settings, including large pragmatic trials in critically ill adults (e.g., DEVICE) [6]. However, VL performance can degrade sharply when the camera lens is obscured by blood or emesis, often forcing device removal, cleaning, and repeated attempts - exactly when time to successful intubation matters most.

This report presents a simple concept for a VL blade modification intended to address two common themes simultaneously: 1) Lens contamination that prevents visualization, and 2) Peri‑intubation oxygen desaturation during apnea.

## Technical report

Device concept and technical description

Unlike existing lens‑clearing VL blades or oxygenation laryngoscopes, this design uses a single integrated gas lumen intended to provide both targeted lens clearing and apneic oxygenation. This device would be designed to be used in prehospital, emergency departments, and possibly ICU settings for trained airway operators.

Design Objective and Elements

The device is a video laryngoscope (VL) blade concept with two intended functions: a primary function to prevent or rapidly clear camera lens contamination by delivering a directed gas jet across the lens, and a secondary function to provide apneic oxygenation during laryngoscopy through the same gas pathway. The blade geometry preserves the overall form factor and technique of a standard Macintosh-style VL blade, with the design also adaptable to other common geometries (e.g., Miller-style). A dedicated internal gas lumen is integrated within the blade, running as an internal channel that terminates near the distal lens region. At the distal end, the outlet/nozzle is oriented to direct a high-velocity jet tangentially across the lens surface, with the intent of shearing liquid films and displacing droplets to mitigate occlusion. The proximal connection is intended to interface with a standard oxygen source (or compressed air) using common clinical fittings and external tubing.

Operational Concept and Failsafe

Continuous low-to-moderate flow may help reduce fogging and prevent gradual lens film formation, while brief high-flow delivery may enable active clearing when the lens becomes visibly obscured. Because this is a conceptual report, specific operating parameters are intentionally not specified; instead, flow rates, nozzle diameter, and jet angle are identified as variables requiring empirical optimization.

If the gas source is unavailable or becomes disconnected, the blade is intended to remain usable as a conventional VL blade without reliance on the gas pathway. In addition, the system is designed to function with either oxygen or medical air, providing flexibility in settings where oxygen conservation is a priority.

Anticipated Clinical Use Cases

This integrated approach is intended to be most useful in scenarios where both visualization loss and hypoxemia risk are high, such as upper gastrointestinal hemorrhage with hematemesis, major facial or oropharyngeal bleeding, trauma with substantial blood and secretions, and cardiac arrest or peri-arrest states where regurgitation risk is elevated.

Importantly, the device is not intended to replace suction-based decontamination strategies (e.g., SALAD). Rather, it is designed to complement suction by directly addressing a common video laryngoscopy failure mode: camera lens obscuration.

**Figure 1:**
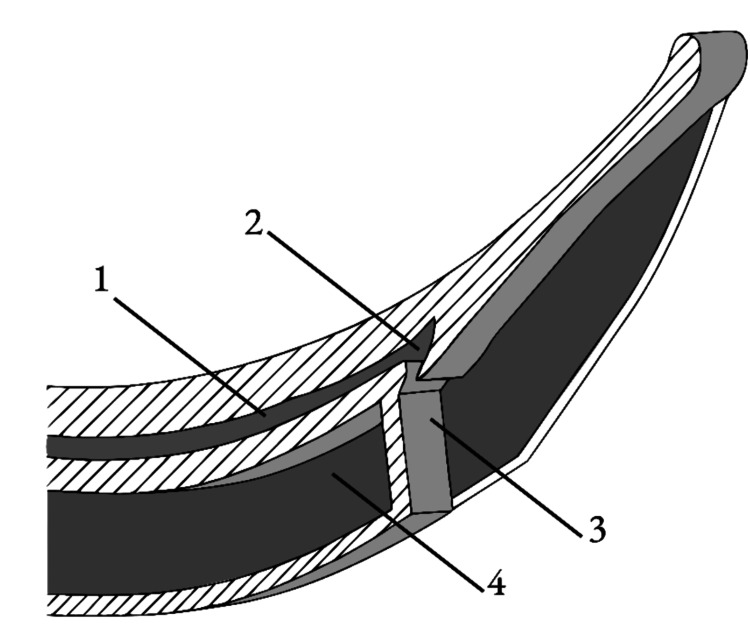
A possible configuration of the device peering inside with a sagittal cut. The device shows a side cross-sectional schematic of the laryngoscope illustrating airflow trajectory toward the camera window. (1) Primary air channel from the main oxygen source. (2) Exit port of the air channel directing flow onto the external video lens. (3) External video lens. (4) Channel housing the video camera or fiberoptic system. Image credits: Kevin Malone, using Autodesk Fusion and Inkscape software

**Figure 2:**
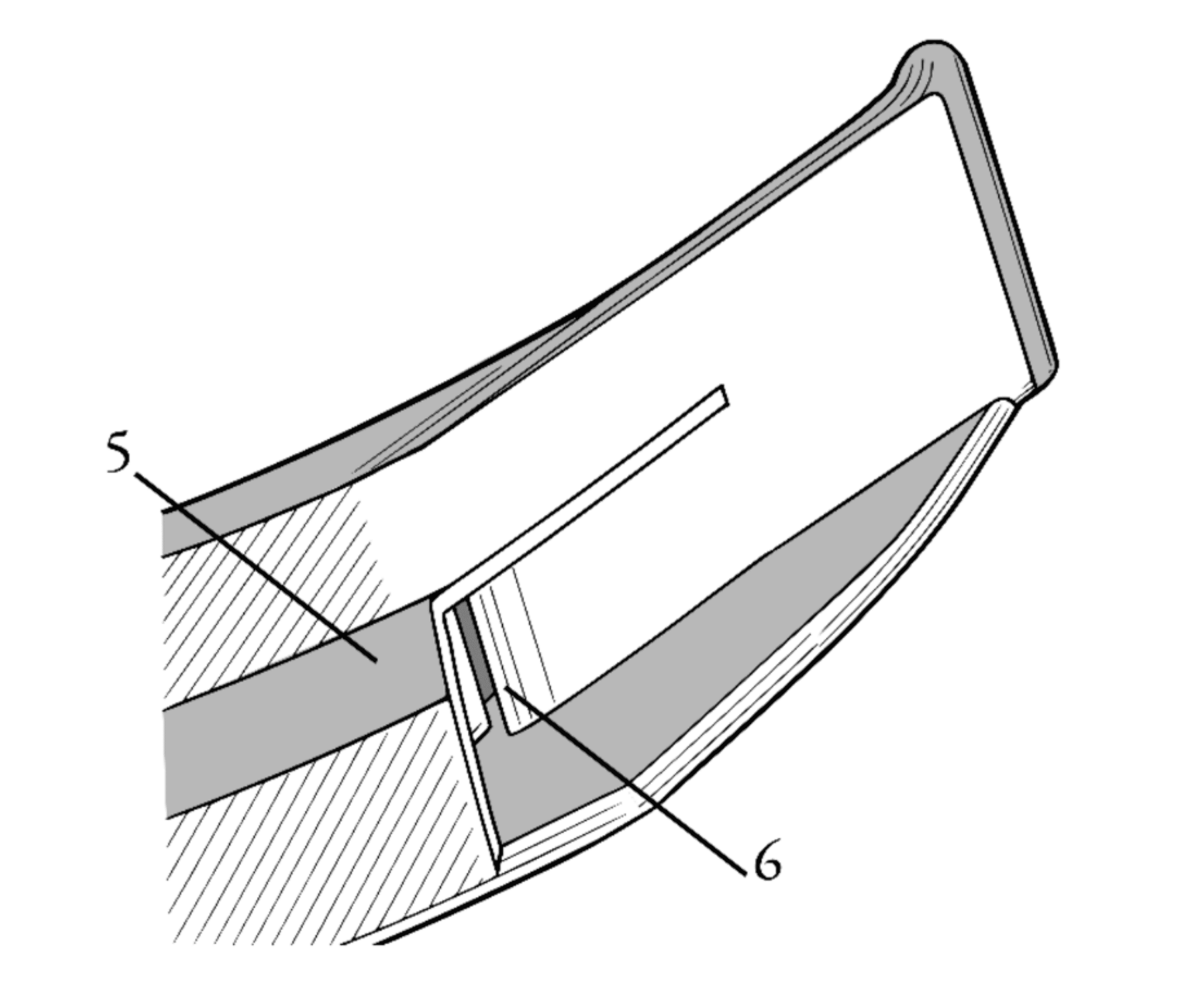
A view of a possible configuration of the device when viewed inferiorly and obliquely. (5) External surface of the camera channel structure showing a non-sectioned view. (6) Inferior view of the air exit nozzle directed at the external video lens. Image credits: Kevin Malone, using Autodesk Fusion and Inkscape software

**Figure 3:**
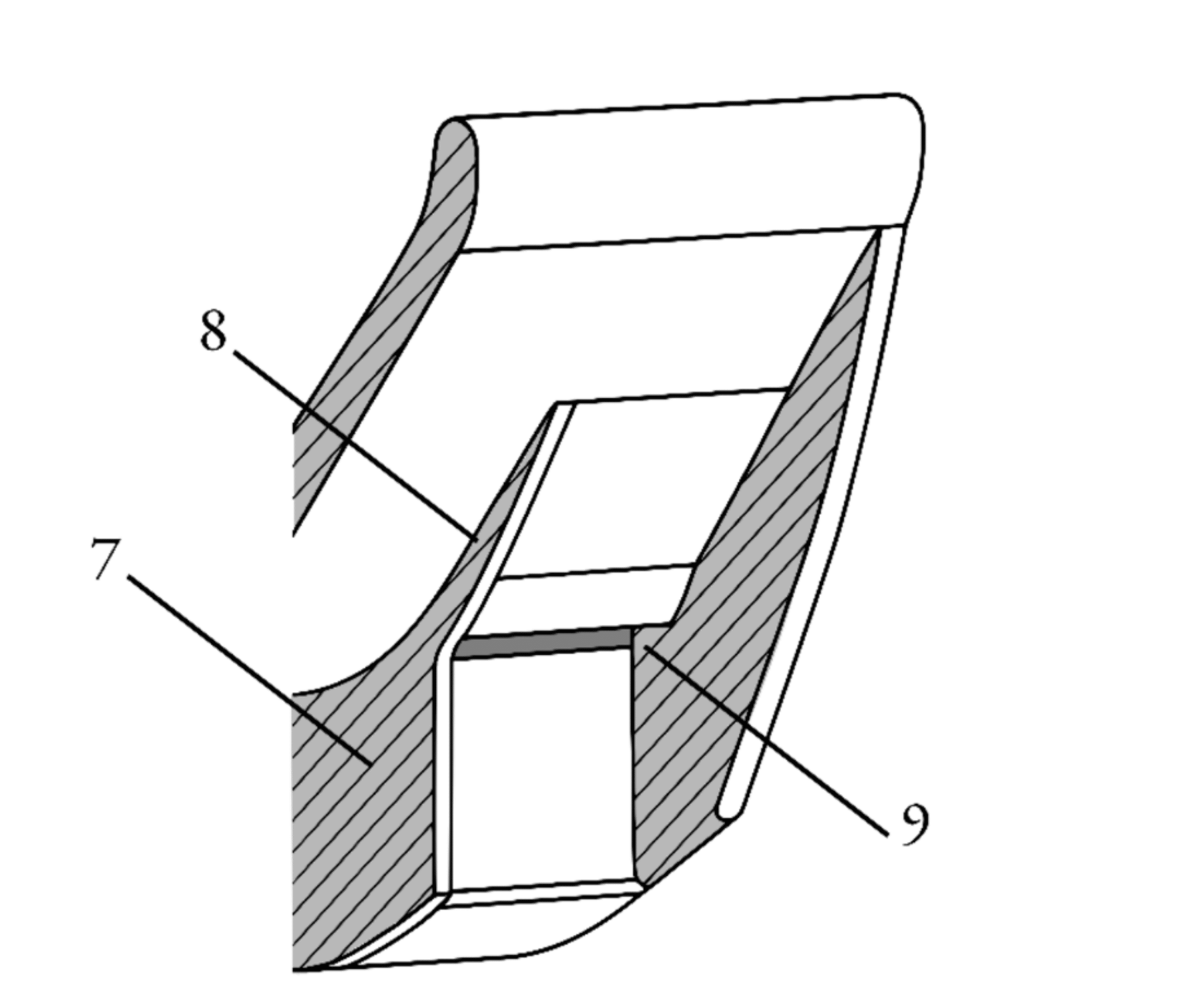
Angled external view with an optional airflow-guiding structure. Viewed somewhat inferiorly and laterally. (7) External housing of the video scope channel. (8) Optional fin or fan-like structure to enhance airflow redirection down the external video lens. (9) Additional perspective view of the air exit opening or nozzle aperture targeting the external video lens surface. Image credits: Kevin Malone, using Autodesk Fusion and Inkscape software

**Figure 4:**
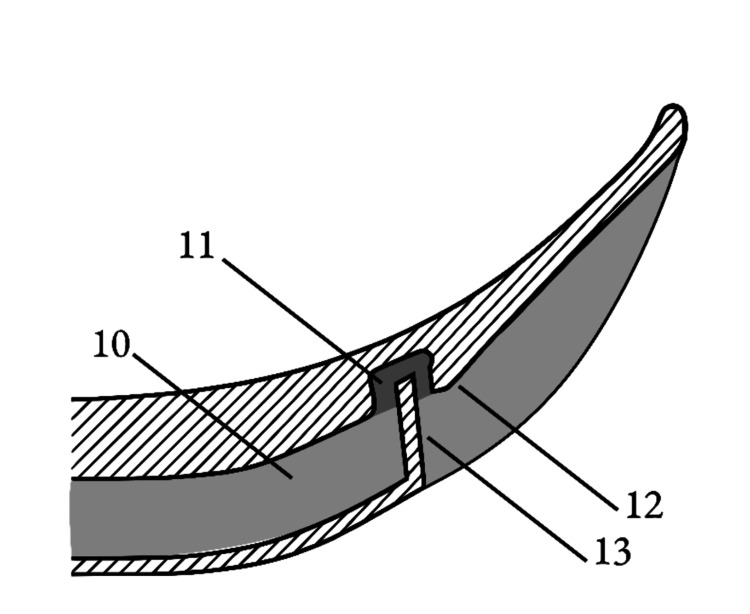
Alternative cross section airflow configuration utilizing empty space in video channel. (10) Open channel intended to house the video camera component. (11) Airflow path integrated around or within the video channel, exiting adjacent to the protective external video lens. (12) Optional perpendicular fin to further direct airflow vertically across the viewing surface. (13) External video lens. Image credits: Kevin Malone, using Autodesk Fusion and Inkscape software

**Figure 5:**
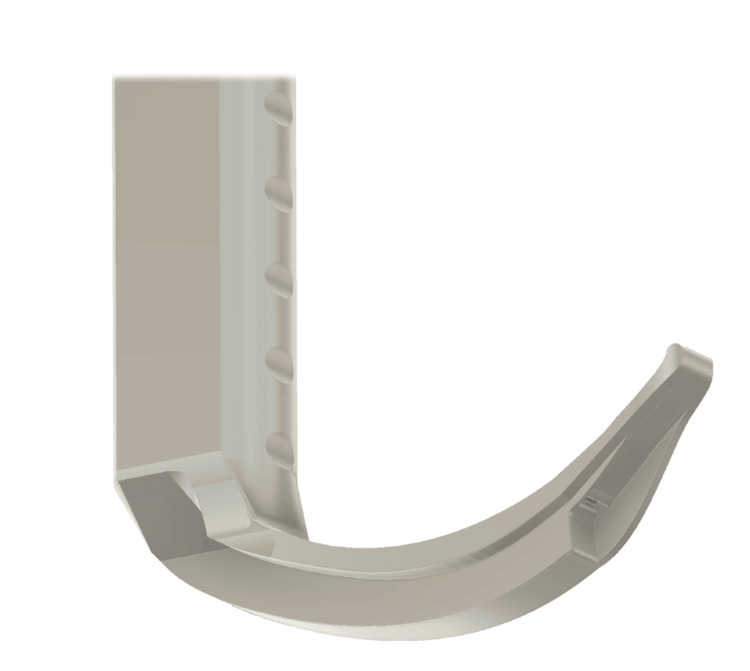
A 3D render of the possible orientation of the device. Image credits: Kevin Malone, using Autodesk Fusion software

The following figures (Figures 1-5) are illustrations of various embodiments of the temple laryngoscope. Components are labeled numerically for reference.

## Discussion

Background and prior approaches

Current best practice in massively contaminated airways emphasizes proactive suctioning and operator technique. The Suction‑Assisted Laryngoscopy and Airway Decontamination (SALAD) approach formalizes simultaneous suction + laryngoscopy and addresses limitations of standard suction practices in heavy contamination [2]. While suction remains essential, visualization failure can still occur when a contaminant contacts the VL lens.

Prior Lens‑Clearing and Anti‑Obscuration Strategies

Lens contamination has been addressed by: removing the laryngoscope and wiping the lens (time‑consuming, increases attempts), improvised saline or built-in flush setups, or purpose‑built lens‑clearing systems [7].

A lens‑clearing VL design has been reported in simulated settings (letter‑level publication), suggesting potential time advantages for experienced users in manikin models [8].

Prior Apneic Oxygenation During Laryngoscopy

Apneic oxygenation via nasal cannula has been evaluated in critically ill intubations, including randomized and meta‑analytic work [9,10]. More proximal oxygen delivery strategies (pharyngeal or laryngeal oxygen insufflation) can extend time to desaturation in experimental and clinical settings, including pediatric trials.

Prototype oxygenation laryngoscopes (oxygen delivery through a laryngoscope channel) have shown promising results in technical simulation, maintaining higher oxygen concentrations in simulated lungs compared with nasal approaches [11].

Oxygenating Laryngoscope

Summary: Lavern describes an “oxygenating laryngoscope” in which removable tubing can be secured along the blade (e.g., by clips) to deliver oxygen toward the airway during laryngoscopy. The patent emphasizes delivering oxygen directly into the upper airway/tracheal region during laryngoscopy and also contemplates suctioning “undesirable fluids” via the tubing. The design intent is to add oxygen delivery (and potentially suction capability) while minimally altering a conventional laryngoscope form factor [12].

How the present concept differs: The present concept is a video laryngoscope blade that uses a directed, high‑velocity gas jet across the camera lens as the primary function (to prevent/clear lens occlusion in a soiled airway), with apneic oxygenation as a secondary function via the same lumen. Lavern’s design is not framed around VL lens occlusion or lens‑directed jet mechanics [12].

Dual‑Use Laryngoscope - Bench/Simulation

Summary: Mitterlechner et al. evaluated a noncommercial “dual‑use” laryngoscope incorporating an internal lumen for oxygen insufflation into a simulated laryngeal space. In their simulation model, oxygen delivered via the device maintained higher oxygen concentrations in a test lung than oxygen insufflation via nasal prongs, supporting the physiologic plausibility of deep laryngeal oxygen insufflation as an apneic oxygenation strategy [13].

How the present concept differs: This work targets oxygenation during apnea but does not address VL lens occlusion in heavily contaminated airways. The present concept explicitly couples oxygen delivery with lens‑clearing/occlusion prevention by shaping and orienting the distal outlet to sweep across the lens [13].

Deep Laryngeal Oxygen Insufflation During Laryngoscopy in Children - Randomized Trial

Summary: Steiner et al. studied deep laryngeal oxygen insufflation during pediatric laryngoscopy, comparing conventional direct laryngoscopy (DL) with oxygen insufflation during DL and oxygen insufflation during video laryngoscopy. They reported substantially slower desaturation slopes with oxygen insufflation compared with conventional DL, consistent with a clinically meaningful extension of “safe apnea time” during laryngoscopy in children [14].

How the present concept differs: This trial evaluates oxygen insufflation (physiology and desaturation kinetics) but does not address the distinct VL failure mode of camera lens obstruction by blood/emesis. The present concept is specifically designed for the soiled airway, where visualization fails due to lens contamination, and uses a dedicated lens‑directed jet to maintain a usable image during active contamination [14].

Oxygenation Laryngoscope - Simulation Model

Summary: Herff et al. evaluated deep laryngeal oxygen insufflation using an “oxygenation laryngoscope” in a controlled simulation setup and reported that oxygen concentration in the test lung remained high in the oxygenation‑laryngoscope condition (reported near mid‑90% range in their model), compared with conditions without equivalent distal oxygen delivery. This supports the feasibility of oxygen delivery closer to the glottis as an apneic oxygenation method in simulation [15].

How the present concept differs: The study’s primary purpose is oxygenation maintenance; it is not a lens‑decontamination system for VL. The present concept integrates oxygen delivery with a lens‑shearing jet intended to prevent and reverse lens film formation and droplet occlusion in the presence of blood/emesis [15].

Modified Macintosh Blades With Oxygen Line Position Variants - Bench Model

Summary: Wetsch et al. investigated apneic oxygenation using modified Macintosh blades with oxygen delivery lines attached at different positions (e.g., tip vs. mid‑blade vs. more proximal). In a bench/lung model, more distal oxygen delivery maintained oxygenation more effectively than proximal placement, which still outperformed room air but did not fully prevent entrainment. The work reinforces that delivery location matters for maintaining a high oxygen fraction at the laryngeal inlet [16].

How the present concept differs: These modified blades focus on oxygen delivery position, not on mitigating VL lens obstruction. The present concept uses a distal, angled outlet with the intent to generate a tangential jet across the camera lens, explicitly optimizing for real‑time visualization in contaminated airways while simultaneously enabling apneic oxygenation [16].

Review/Technical Discussion of Apneic Oxygenation With Blade‑Lumen Insufflation

Summary: Pratt’s practical review describes apneic oxygenation strategies and explicitly notes laryngoscope blade designs incorporating an internal lumen for oxygen insufflation during laryngoscopy. This reflects broader recognition that oxygen delivery can be integrated into laryngoscopy equipment beyond nasal cannula approaches [11].

How the present concept differs: The review discusses the apneic oxygenation conceptually and does not propose a VL lens‑directed clearing jet. The present concept is engineered around visualization failure in the soiled airway (lens occlusion) as a primary design target, using gas flow both for lens maintenance and oxygenation [11].

Low‑Cost Modified Macintosh Video Laryngoscope for Hypoxemic COVID‑19 Patients

Summary: Mustahsin et al. report a low‑cost video laryngoscope created by modifying a standard Macintosh blade, motivated by equipment scarcity and infection‑control concerns during the COVID‑19 pandemic. Their abstract emphasizes feasibility, usability without additional training, and substantial cost reduction relative to commercial VL devices [17].

Additional descriptions associated with this report reference deep laryngeal oxygen insufflation as a means of mitigating peri‑intubation desaturation in hypoxemic patients.

How the present concept differs: This report is primarily about accessibility and cost of VL capability (and potentially oxygen insufflation), not about lens‑contamination mechanics in massively soiled airways. The present concept is designed specifically to prevent/clear lens occlusion by blood/emesis via a targeted jet, with apneic oxygenation as a secondary function [17].

Portable Video Laryngoscope With Oxygen/Infusion and Suction Capabilities

Summary: Smith and Smith describe a handheld, probe‑based portable video laryngoscope with camera visualization and multiple channels/ports. The device includes an infusion port that can be connected to oxygen tubing; the patent explicitly states oxygen flow can push back soft tissues and flow over the camera lens to help maintain an unobstructed view and reduce secretion accumulation. The port can also accept syringes for irrigants/medications, and the system includes suction capability for secretion/blood removal [18].

How the present concept differs: While Smith and Smith already articulate the idea of gas flow over a lens, the present concept differs by targeting a Macintosh‑style VL blade and by specifying a distal jet/nozzle geometry oriented tangentially across the VL lens as the primary intervention for soiled‑airway lens occlusion. The present concept is also framed as a low‑complexity blade modification intended to preserve familiar VL technique and incorporate burst/continuous flow modes for lens clearing [18].

Video Laryngoscopy Device With Air‑Based Lens Cleaning Mechanism

Summary: Newcomb and Gonzalez describe a disposable video laryngoscopy device that includes structural features intended to reduce debris adherence (e.g., a protective cover shield) and a camera lens cleaning mechanism configured to inject air toward the image sensor/lens region. The patent describes a push‑button‑activated system with an air reservoir, nozzle, tubing terminating near the image sensor, and check valves/filters to regulate one‑way flow and refill the reservoir [19].

How the present concept differs: The Newcomb design focuses on an integrated air‑burst cleaning subsystem for the camera region, but it is not presented as an oxygenation strategy, nor is it framed around apneic oxygenation during laryngoscopy. The present concept explicitly uses a clinically available gas source (oxygen or medical air) through an internal blade lumen to achieve both lens clearing and apneic oxygenation via the same pathway, with the distal outlet oriented to sweep the lens [19].

IntuBlade Lens‑Clearing Video Laryngoscope - Simulated Evaluation + Commercial Lens‑Spray Mechanism

Summary: Napier and Zitek report (letter‑level publication) a simulated evaluation in which experienced users achieved decreased time to intubation with a “lens‑clearing video laryngoscope”; however, the PubMed record provides no abstract, limiting extraction of technical details and endpoints from that source alone [8].

Commercial descriptions of the IntuBlade system describe a built‑in lens spray mechanism, commonly described as a saline spray/flush used to clear debris from the camera lens during intubation [8,20].

How the present concept differs: IntuBlade’s lens clearance is described as a fluid (saline) spray/flush mechanism and is not designed to provide apneic oxygenation via the blade. The present concept instead uses a gas jet (oxygen or air) intended to clear and prevent lens contamination while simultaneously providing apneic oxygenation, reducing added components (e.g., syringes/flush fluid) and aiming to maintain oxygenation during prolonged laryngoscopy in physiologically unstable, contaminated airways [8,20].

Fluid/Gas Dynamics for Improving Endoscopic Visibility, Including Intubation Scopes

Summary: Mejia describes modifications to endoscopic apparatus using fluid and gas dynamics to improve visibility, including scopes intended for orotracheal intubation. The patent explicitly frames the problem of difficult airways, where blood/secretions obscure the view and describes directing air or fluid flow over a lens within the scope; it also discusses limitations of purely longitudinal injection and references distal port arrangements intended to improve delivery directionality [21].

How the present concept differs: Mejia is a broad “scope” patent spanning multiple endoscopic configurations and fluid/gas routing concepts, rather than a VL blade optimized for the rapid emergency intubation technique. The present concept narrows the solution to a video laryngoscope blade with a single internal gas lumen terminating in a lens‑sweeping jet and explicitly couples lens clearing with apneic oxygenation using typical clinical gas sources [21].

Laryngoscope Guide and Related Method of Use - Guidance Conduit for Introducer/Bougie

Summary: Rosenthal describes a laryngoscope guide incorporating a conduit/bore through which an introducer (e.g., stylet/bougie‑like element) passes and can be shaped by the conduit geometry. The patent emphasizes aligning the introducer’s advancement axis with the user’s visual field (including video/monitor viewing) to facilitate steering the introducer toward the glottic opening and into the trachea [22].

How the present concept differs: This invention addresses tube/introducer guidance and alignment, not lens contamination or oxygenation. The present concept targets a different failure mode - visualization loss due to camera lens soiling - and adds apneic oxygenation via a gas lumen/jet rather than a guidance conduit for an introducer [22].

Gap: To our knowledge, no existing video laryngoscope blade design simultaneously addresses the dual challenges of lens occlusion and apneic oxygenation through a single, integrated gas lumen optimized for both functions. Our concept bridges this gap, targeting a primary failure mode of VL in the soiled airway while leveraging the same mechanism to mitigate a primary physiological risk.

Safety considerations and potential risks

Any lens-clearing jet used in a contaminated airway introduces non-trivial safety questions that must be addressed before clinical use, including aerosolization risk, airway/aspiration dynamics, oxygen-related fire considerations, and human factors. A high-velocity jet could aerosolize secretions or blood, so directionality (i.e., sweeping across the lens and toward the airway rather than out of the mouth), PPE expectations, and practical flow limits should be systematically evaluated. Additional upper-airway gas flow could also theoretically affect gastric insufflation or distally displace contaminants, which warrants simulation and bench testing; preliminary calculations suggest distal nozzle pressure would not exceed 10-15 cm H₂O, a range generally considered safe for upper-airway insufflation and well below pressures associated with gastric insufflation during positive-pressure ventilation, but this assumption should be empirically validated [23]. There may be some instances where this device may have difficulty or not be able to cleanse the lens, such as in situations where there are highly viscous or solid materials; however, more studies are needed.

Because oxygen delivery increases oxidizer concentration, the design also implicates oxygen-enriched-environment precautions; although typical ED airway management has few ignition sources, operative settings and electrocautery-adjacent workflows require heightened caution. Finally, the system must be engineered for usability: controls should be simple and intuitive, and added tubing should not meaningfully increase setup time or cognitive load during high-stress airway management; even if the mechanism is conceptually straightforward, it still introduces extra tubing and an additional control interaction that must be accounted for in human-factors testing.

Proposed development and validation pathway

Because this is currently a conceptual design, a stepwise evaluation pathway is proposed that progresses from controlled bench testing to simulation, then to carefully monitored clinical use, and finally to effectiveness testing if feasibility and safety are established. Initial work would use a benchtop lens-contamination model with standardized application of fluids spanning a range of viscosities/opacity (e.g., simulated blood, simulated emesis) to quantify time-to-clear and residual visibility while varying jet angle, outlet/orifice size, and flow. Next, high-fidelity simulation and manikin studies would compare standard VL to the lens-clearing, oxygenating VL in standardized “soiled airway” scenarios, using endpoints such as time to best view, time to intubation, first-pass success, and the number of device removals for lens cleaning. If these data support feasibility and acceptable safety signals, a pilot clinical feasibility study could follow with limited deployment under protocolized conditions and strict safety monitoring. Contingent on successful completion of these phases, a subsequent clinical effectiveness trial could evaluate patient-centered outcomes such as hypoxemia incidence, number of attempts, and peri-intubation complications.

Cost and Manufacturability Considerations

For prototyping, additive manufacturing can produce low-cost iterations for iterative testing. However, any clinical deployment would require appropriate materials, sterility/processing considerations, and regulatory pathway planning. Injection molding and ultrasonic welding processes used in current disposable VL blades may be adaptable to incorporate an internal lumen with minimal added mechanical complexity, allowing this device to be commercially available. Therefore, cost additions are likely to be minimal to near zero, unless the addition of these channels leads to manufacturing errors.

Limitations

This report describes a concept; it is not yet supported by clinical outcome data. Lens clearing may be less effective against highly viscous particulate contaminants without concurrent suction. The optimal flow/nozzle geometry is unknown and requires experimentation. Safety questions - as mentioned above - (aerosolization, aspiration dynamics) must be empirically evaluated. The jet will be directed tangentially across the lens and towards the glottis, minimizing aerosolization towards the operator. Simulation studies should be done to quantify aerosol generation using fluorescent tracer particles and high-speed imaging. In clinical use, the device should be considered an aerosol-generating procedure mandating appropriate PPE. This is a conceptual device and not FDA cleared; it is currently described for research and development purposes.

## Conclusions

By transforming the video laryngoscope blade from a solitary visualization tool into an active airway management platform, this device has the potential to guide improvements in first-pass success strategies in the most challenging intubations. A VL blade with an integrated gas lumen delivering a targeted jet across the camera lens represents a plausible low‑complexity approach to a persistent failure mode in the contaminated airway. By coupling lens‑occlusion prevention/clearing with apneic oxygenation, this concept aims to reduce interruptions and repeated attempts during the most time‑sensitive intubations. If validated through bench, simulation, and clinical testing, the approach may improve airway management in scenarios where seconds determine hypoxic injury risk.
